# Diagnosis of Cutaneous Larva Migrans using Point of Care Ultrasound

**DOI:** 10.24908/pocus.v9i1.17470

**Published:** 2024-04-22

**Authors:** Daniella Lamour, Robert A Farrow, Jean Pierre, Daniel Puebla, Paul Khalil

**Affiliations:** 1 Nicklaus Children's Hospital Miami, FL USA; 2 Florida Atlantic University Boca Raton, FL USA; 3 Mount Sinai Medical Center Miami, FL USA

**Keywords:** cutaneous larva migrans, soft tissue ultrasound, point of care ultrasound

## Abstract

Larva migrans is a cutaneous parasitic infection that occurs when an immature hookworm larva inadvertently penetrates the dermis of a human, typically on the extremities. Traditionally, a clinical diagnosis is made when a tortuous/serpiginous eruption is seen superficially in the skin with complaints of intense pruritus. Point of care ultrasound (POCUS) is a useful diagnostic tool for soft tissue complaints in the emergency department (ED). We describe a case of an 18-year-old woman who presented to the ED with foot pruritis four days after walking on the beach barefoot. POCUS examination revealed several motile structures in the dermis of the patient’s foot, confirming our suspicion of cutaneous larva migrans. The patient was then placed on an oral anthelmintic and her symptoms resolved shortly after.

## Introduction

Cutaneous larva migrans (CLM) is a common zoonotic infection that occurs when the filariform larva of the hookworm penetrates the epidermis of a human’s skin. The adult hookworm usually lays its eggs in their natural hosts, cats and dogs 1. The most common species of these hookworms are Ancylostoma brasiliense (cat hookworm) and Ancylostoma caninum (dog hookworm), and are commonly seen in the southern United States, the Caribbean, and South America 1. The prevalence of hookworms has been reported up to 8% in certain populations, most commonly in children 2. Risk factors include a young age (10-14 years old), male sex, resource-poor region, and walking barefoot 2. These organisms thrive in warm and moist environments, and are conventionally present amongst travelers in tropical regions. 

Humans can become inadvertent hosts, typically from walking barefoot and accidentally stepping on contaminated animal feces infested with the hookworm. The hookworm is unable to travel into deeper layers of the skin to complete its life cycle in a human’s gastrointestinal tract due to a deficiency in the collagenase enzyme 3. Thus, they migrate throughout the epidermis, creating the classic superficial serpiginous tracks which may last a few weeks to months, and oftentimes fully resolve without treatment 4. Without an objective diagnostic tool, this classically remains a clinical diagnosis. POCUS is a well-established tool which has been shown to increase diagnostic accuracy in soft tissue infections in pediatric emergency medicine 5. The most common uses of soft tissue ultrasound (US) include cellulitis, abscesses, and foreign bodies. When there is an increased suspicion of a foreign body, US can be used to detect, localize, and potentially extract it 6. CLM, a common parasitic infection which may be considered a foreign body, has scarcely been reported to be diagnosed using POCUS. We present a case of CLM confirmed on POCUS in a tertiary pediatric emergency department (ED). 

## Case Presentation

An 18-year-old woman with a history of Diabetes mellitus type 1 presented with pain, itching, and swelling of the left heel for one day. Her symptoms worsened with weight-bearing and improved with rest. She stated she was at the beach four days prior but denied any injury or having stepped on anything sharp. Her vital signs were normal. The physical examination was normal except for the soft tissue exam which revealed localized swelling with erythema on the heel that was mildly tender to palpation without fluctuance, induration, or swelling. POCUS examination was performed to further examine the area of interest and to evaluate for the presence of a foreign body or abscess. It revealed a serpiginous motile structure in the dermal layer of the foot, confirming the diagnosis of CLM. The patient was given a prescription for a one-time dose of Ivermectin with improvement of symptoms. 

### POCUS Findings

**Figure 1:**
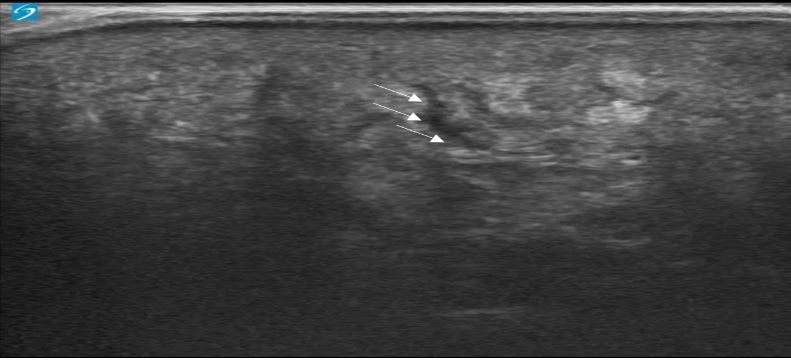
An anechoic motile serpiginous parasite (arrows) detected while tunneling through the epidermis.

**Figure 2:**
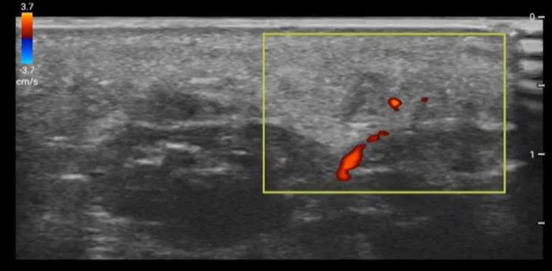
Color Doppler was applied near a second anechoic track (arrows). Lack of color flow confirmed anechoic lesion was not a vascular structure.

A high-frequency linear probe was used to evaluate the plantar aspect of the patient’s foot. The images revealed several small linear hyperechoic lesions in the subcutaneous layers of the foot exhibiting serpiginous motility (Figure 1). The subcutaneous tissue shifted throughout the migration process. Color Doppler was applied to confirm the moving echogenic lesions were not vasculature structures (Figure 2). The contralateral foot was also scanned for comparison and there was an absence of these motile structures in the epidermis. 

## Discussion

Cutaneous larva migrans can be diagnosed solely on a patient’s clinical presentation, however, additional studies may help confirm the diagnosis. Serological tests, such as enzyme-linked immunosorbent assay, can detect specific antibodies against the causative parasites 7. As well, skin biopsy can be performed to identify the presence of larvae in the skin, but it may be inconclusive, rather invasive, and unnecessary. In other cases, imaging studies like X-rays may be employed to visualize the larvae in visceral tissues 8. Optical coherence tomography has been reported to be an effective minimally invasive tool to rapidly detect CLM 9. However, it is not widely available in the ED and is rarely used outside of an ophthalmology office. Although dermoscopy has been used to detect these cutaneous parasites, most EDs do not carry these devices. In a recent study, reflectance confocal microscopy confirmed a larva burrow, described as a hyporeflective disruption of the normal honeycomb pattern in the epidermis 6. As one can imagine, these devices are more commonly found in dermatology offices and are not typically stocked in most EDs. Interestingly, a high-frequency US was also utilized in that same study. A cylindrical mass and shadowing were revealed which the authors believed may have corresponded to the parasite and larva burrow 6.

There are limited studies demonstrating cutaneous larva migrans with minimally invasive imaging tools in pediatric patients in the ED. In contrast to other imaging modalities, POCUS is a valuable noninvasive portable imaging tool, available in most EDs. It has many clinical applications, including distinguishing between different soft tissue complaints. This case report features POCUS used to detect cutaneous larva migrans. We highlighted motile hyperechoic lesions in the epidermal layer of the patient’s foot, utilizing a high-frequency linear transducer. When these hookworms tunnel through the skin, their paths are highlighted as anechoic tracks amid the echogenic base representing the dermis. Unfortunately, the individual larvae were not detected. While visualizing the actual larva may require more effort and time, the parasitic tracks can be detected via US, as seen with our patient. This technique may prove to be even more clinically useful as another case demonstrated that suspected larva diagnosed via US imaging, were normal soft tissue 6. As more POCUS implementation is used by clinicians who suspect CLM, there will be more images available to compare our images against. This will perhaps give insight to diagnostic criteria for CLM.

Our patient complained of swelling and pruritus on the sole of her foot upon her presentation to the ED. Although the typical presentation of CLM consists of tortuous, pruritic, and erythematous lesions, presentation and symptoms are variable and CLM may be easily misdiagnosed due to lack of recognition and/or confidence in the diagnosis. As presented in our case, our patient did not have the classic serpiginous lesions, which may have led to a misdiagnosis and inappropriate treatment. POCUS may serve as a useful confirmatory tool prior to initiating treatment of CLM with oral anthelmintics. The first line recommendation for anthelmintics is a one-time dose 200 mcg/kg oral Ivermectin, which usually results in a 100% cure rate. If necessary, Albendazole may be used second line. Topical anthelmintics like Thiabendazole may also be effective if infection is local, but has been reported to have a poor eradication rate. Our patient received one dose of Ivermectin with full resolution of her symptoms. As CLM is very responsive to treatment, early and accurate diagnosis via US evaluation is likely clinically valuable.

## Conclusion

The diagnosis of CLM historically has been made by history and physical exam. Other suggested imaging modalities are either cost restrictive, insufficient, or not readily available. In this case, POCUS helped confirm the diagnosis and is not hindered by the same limitations of other imaging modalities when assessing for CLM. Further studies are needed to confirm the utility of POCUS to aid in the diagnosis of parasitic infections. 

## Disclosure Statement

All authors declare no relevant financial relationships.

## Patient Consent

The authors’ institutional research ethics board does not require the obtainment of informed consent for the preparation of de-identified case reports.
